# Wavelet-Assisted Adaptive EKF Phase Shift Estimation Approach for Motion-Induced Error Compensation

**DOI:** 10.3390/s26051735

**Published:** 2026-03-09

**Authors:** Xin Lai, Qiushuo Yu, Zhenyi Chen

**Affiliations:** 1 School of Mechanical and Electrical Engineering, Southwest Petroleum University, Chengdu 610500, China; 202322000550@stu.swpu.edu.cn; 2 School of Sciences, Southwest Petroleum University, Chengdu 610500, China; 202099010109@swpu.edu.cn

**Keywords:** phase estimation, extended Kalman filter, state-space model, wavelet transform, motion-induced compensation

## Abstract

Phase-shifting profilometry (PSP) suffers from motion-induced phase-step variations in dynamic scenes. The breakdown of the fixed phase shift assumption results in issues such as ripples, distortions and accuracy decline in PSP systems. To reduce motion-induced phase errors, we propose a wavelet-assisted adaptive extended Kalman filter (WAEKF) to estimate varied pixel-wise phase shift. A wavelet-based strategy is presented to extract an initial spatial carrier frequency at each row from fringe patterns for EKF estimation. A state-space model employing the quadrature phase component and carrier frequency is established in this paper. The unknown phase shifts can be evaluated by using a forward–backward filter. Experiments show that the proposed method can acquire an accurate initial carrier frequency and phase shift map, which effectively reduces 3D reconstruction error and can be extended to N-step PSP systems.

## 1. Introduction

Phase-shifting profilometry (PSP) is a widely used structured-light technique for high-accuracy, non-contact three-dimensional (3D) shape measurement. In PSP systems, sinusoidal fringe patterns are projected and demodulated to retrieve the 3D shape of the object. This technique has found widespread application in industrial inspection, robotic perception, and biomedical measurement [1,2,3,4,5]. In dynamic scenes, phase shift deviations are induced from the object motion in the projected fringe patterns. Crucially, these deviations typically exhibit non-uniform, pixel-wise spatial variations across the object [6]. Preset parameters of the PSP method undergo variations, including the carrier frequency and phase shift. Such variations lead to errors in the unwrapped phase and continuous phase in the reconstructed 3D shape.

In practical measurements, phase shift uncertainty mainly arises from two sources: the projection devices, such as nonlinearity or timing errors in digital light projectors (DLPs) [7,8], and the object motion during dynamic measurements, including translation, rotation, or non-rigid deformation, which induces additional phase shift deviations and phase errors [9,10,11,12]. Such phase errors degrade the reliability of conventional PSP algorithms in dynamic scenes and often give rise to severe motion artifacts in the reconstruction. Researchers reduce motion-induced errors by increasing the acquisition rate of the projection and image system [13,14], which effectively reduces errors in the phase shift introduced by the projector. Fringe-based 3D imaging frameworks have also been explored to cut down the required number of projected images [15,16]. A variety of compensation methods have been proposed to address this issue, including object-tracking approaches [17,18], Fourier-assisted PSP methods [19,20], iterative least-squares algorithms [21,22], Hilbert transform-based methods [23,24,25], the statistical property compensation strategy [26], the deep-learning-based approach [27,28,29], and the motion prediction method [30]. These studies can be broadly divided into pixel-wise and region-wise estimation methods.

Pixel-wise compensation methods have been introduced to correct motion-induced errors. Guo et al. [31] presented a real-time average-compensation scheme for four-step phase-shifting profilometry. The inverse-splitting dual three-frame strategy was developed to generate two phase maps within the same framework, and the average phase is used to compensate for motion error. This method suffers from the inherent limitations of Fourier fringe analysis method. Guo et al. [32] further proposed a generalized phase shift deviation estimation method for N-step phase-shifting profilometry, N-step fringe images are regrouped according to an image-reuse strategy, and phase shift deviations are obtained from the differences between adjacent phase maps. Yu et al. [33] introduced a quasi-pixel-wise compensation scheme tailored to the four-step phase-shifting profilometry. By taking advantage of the periodic nature of the fringe patterns to estimate the phase steps, this approach markedly suppresses motion-induced ripples and remains effective for objects undergoing non-rigid deformation. Zhang et al. [34] proposed a binomial self-compensation (BSC) strategy to iteratively compensate motion-induced errors at the pixel level across multiple frames. Li et al. [28] developed a pixel-wise motion compensation approach based on deep learning for rapid 3D reconstruction of dynamic scenes. In their framework, a NAS-designed compact network is combined with dual-frequency fringe projection to output the unwrapped phase, thereby realizing fast and accurate 3D shape recovery. However, this data-driven approach requires large training datasets, and its generalization is limited when measurement conditions differ from those in the training data. Jeon et al. [35] reported a pixel-wise approach aimed at reducing motion-induced errors in a motor-driven digital fringe projection system. Their technique employs a motion-sensitive phase-shifting scheme, incorporating motor encoder data and camera–projector pinhole modeling, to correct pixel disparities and phase shift deviations with only three fringe projections. However, this solution is strongly customized to the specific motorized configuration and does not readily adapt to broader motion conditions or alternative hardware designs. Pixel-wise estimation methods provide fine-grained, high-accuracy phase shift compensation and effectively suppress motion-induced artifacts, but some methods may increase computational complexity.

Region-wise compensation methods utilize local information to reduce motion phase errors and then propagate the correction across the entire field. Liu et al. [36] developed a compensation scheme for phase-shifting profilometry, in which object motion is inferred from the differences between two successive 3D frames and the resulting information is used, via a projector pinhole model, to estimate the phase shift errors. Feng et al. [9] introduced an iterative scheme for estimating phase shift deviations within a dynamic three-dimensional measurement framework. In this approach, the phase error is derived from the statistical nature of the fringes, and reliable neighboring pixels are then exploited to refine the phase error correction. Cong et al. [19] introduced a Fourier-assisted phase-shifting profilometry method, the phase shift deviations are compensated by single-frame Fourier transform profilometry using spectral information, which enhances the accuracy of dynamic 3D measurement for rigid motion, but it is not applicable to non-rigid deformation. An extended Kalman filter method was proposed by Lai et al. [37] for phase shift estimation. In this method a local quadratic phase model is utilized to obtain the actual phase step and compensates for the uncertain phase shifts. Subsequently, Lai et al. [38] developed an extended Kalman filter scheme for phase shift estimation that explicitly incorporates background information. In their method, a 2D-VMD-BEMD-based process isolates the background intensity and guides window selection, allowing reliable estimation of actual phase steps under dynamic scenes and leading to more accurate 3D reconstructions. By adopting a local-to-global strategy, the 3D reconstruction process is accelerated and both accuracy and computational efficiency are improved. However, it cannot fully capture the varying phase shifts across all pixels in the field. In dynamic scenarios, the carrier frequency during projection may slightly deviate from the preset value due to object motion, especially during rotation. Therefore, it is necessary to obtain the actual carrier frequency from the captured fringe patterns. Inspired by these, we explore a full-field adaptive EKF estimation approach for phase shift estimation.

To address the full-field phase shift variations induced by object motion in dynamic scenes, this paper presents a wavelet-assisted adaptive extended Kalman filter (WAEKF) to estimate the additional phase shift deviation. A wavelet-based scheme is employed to achieve the carrier frequency for providing an initial value for adaptive EKF estimation. A state-space model constructed from the carrier frequency and quadrature components performs Bayesian recursive estimation. The carrier frequency and wrapped phase can be reliably obtained when the fringe pattern is disturbed by the object motion. Experiments demonstrate that the proposed method effectively estimates the carrier frequency and phase shifts, thereby significantly improving the accuracy of 3D reconstruction in dynamic scenarios. The proposed method focuses on the dynamic measurement of the moving objects, where the acquired fringe patterns are processed offline rather than in real-time. The remainder of this paper is organized as follows: Section 2 describes the principle and framework of the proposed WAEKF. Section 3 presents the experimental validation under the translation and rotation motions. Finally, Section 4 presents the conclusion.

## 2. Principle

### 2.1. Phase-Shifting Profilometry with Unknown Phase-Shift Error

In three-step phase-shifting profilometry, three sinusoidal fringe patterns with nominal phase steps are projected and captured to recover the object phase. Under the ideal assumption, the recorded intensity at pixel in the *k*-th step can be written as [39]

(1) $$I_{k}^{ideal}\left(x,y\right)=I_{A}\left(x,y\right)+B\left(x,y\right)\cos[\phi_{k}\left(x,y\right)+\omega_{k}x+\delta_{k}^{ideal}]\;\;\;\;\;\;\;\;\;\;\;k=1,2,3$$

where $I_{A}\left(x,y\right)$ and $B\left(x,y\right)$ represents the background intensity and modulation, respectively. $\phi \left(x,y\right)$ is the phase of the measured object. $\delta_{k}^{ideal}$ is the fixed phase step, that is, $2\pi \left(k-1\right)/3$ in the standard three-step phase-shifting algorithm. However, actual phase shift often deviates from the fixed value due to the projector, camera, mechanical jitter and motion of objects. Therefore, the actual phase shift $\delta_{k}$ is expressed as

(2) $$\delta_{k}=\delta_{k}^{ideal}+\Delta \delta_{k}$$

where $\Delta \delta_{k}$ is an unknown phase shift deviation which can spatially vary in practical situations.

**Figure 1:**
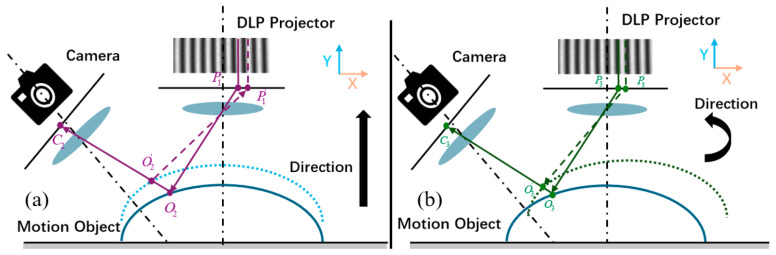
Phase-shift errors induced by the motion: (**a**) translation motion; (**b**) rotation motion.

To explicitly connect phase shift deviations with the carrier frequency employed in this work, let $\Phi_{k}(x,y)=\phi_{k}\left(x,y\right)+\omega_{k}x+\delta_{k}$. For commonly used vertical fringes (varying primarily along x), the captured intensity can be expressed as

(3) $$I_{k}\left(x,y\right)=I_{A}\left(x,y\right)+B\left(x,y\right)\cos\left(\Phi_{k}(x,y)\right)\;\;\;\;\;\;\;\;\;\;\;k=1,2,3$$

where $\omega_{k}=2\pi f_{k}$ is the spatial carrier frequency. In dynamic measurement, object motion undermines the ideal temporal phase-shifting assumption. Specifically, translation or rotation motion causes the phase shift to deviate from its preset value. As demonstrated in Figure 1, this deviation results in motion-induced phase errors. Motion-induced phase errors are inherently pixel dependent and may vary significantly across regions with distinct motion behaviors or geometries.

The wrapped phase of the traditional three-step phase-shifting algorithm can be expressed as

(4) $$\phi_{traditional}\left(x,y\right)=\arctan[\frac{\sum_{n=1}^{N}\left(I_{n}\left(x,y\right)\sin\frac{2\pi \left(n-1\right)}{N}\right)}{\sum_{n=1}^{N}\left(I_{n}\left(x,y\right)\cos\frac{2\pi \left(n-1\right)}{N}\right)}]$$

The wrapped phase obtained by Equation (4) exhibits a decline in reconstruction accuracy due to phase shift deviation. Therefore, it is necessary to estimate the actual phase shift to obtain an accurate wrapped phase. Furthermore, the carrier frequency of the captured fringe patterns deviates from the preset value, and it is necessary to extract it using the wavelet method to provide prior information. A Bayesian inference model is constructed to estimate the quadrature components, which achieves the unknown phase shift. Therefore, we develop a new wavelet-assisted adaptive EKF state-space framework.

### 2.2. Wavelet-Assisted Adaptive EKF State-Space Model

A wavelet-assisted adaptive EKF state-space model is formulated to mitigate the phase bias caused by phase shift deviations in the three-step PSP. The dominant spatial carrier frequency is first estimated via wavelet analysis and incorporated into the state estimation model as a prior, thereby constraining the adaptive EKF prediction. Subsequently, the quadrature components are recursively estimated from each single fringe image, from which the instantaneous phase is obtained to support later phase shift deviation estimation and compensation.

The background component of the fringe pattern, affected by ambient light, reflectance, and sensor non-uniformity, varies over time and biases phase-related estimation by degrading modulation contrast and increasing noise sensitivity. Consequently, the removal of the DC term improves the reliability of 3D reconstruction. We perform carrier estimation row by row of the captured fringe pattern. For each row *x*, the background is removed by the following method.

(5) $$I_{k,y}^{\prime }\left(x\right)=I_{k}\left(x\right)-A\left(x\right),\;\;{\tilde{I}}_{k,y}^{\prime }\left(x\right)=I_{k,y}^{\prime }\left(x\right)-\frac{1}{n}\sum_{x=1}^{n}I_{k,y}^{\prime }\left(x\right)$$

where *k* is the number of pixels in the row. $I_{k,y}^{\prime }$ and ${\tilde{I}}_{k,y}^{\prime }$ denotes the signal without background intensity $A\left(x\right)$. $A\left(x\right)$ can be obtained through the modified 2 + 1 PSP. The continuous wavelet transform of ${\tilde{I}}_{k,y}^{\prime }\left(x\right)$ along the *x*-direction is computed by

(6) $$W_{k,y}\left(\alpha ,u\right)=\int_{-\infty }^{+\infty }{\tilde{I}}_{k,y}^{\prime }\left(x\right)\frac{1}{\sqrt{\alpha }}\psi^{*}\left(\frac{x-u}{\alpha }\right)dx$$

where α and u are the scale and spatial position, respectively. $\psi \left(\cdot \right)$ denotes an analytic mother wavelet. The scalogram magnitude is defined as

(7) $$A_{k,y}\left(\alpha ,u\right)=\left|W_{k,y}\left(\alpha ,u\right)\right|$$

A row-wise dominant-scale selection rule is

(8) $$\alpha_{k,y}^{*}=\arg\max_{\alpha }\left(\frac{1}{N}\sum_{u=1}^{N}A_{k,y}\left(\alpha ,u\right)\right)$$

The generalized Morse wavelet is employed as the mother wavelet to process the 8-bit fringe patterns due to its analyticity and time–frequency localization. The symmetry parameter and the time–bandwidth product of the Morse wavelet are set to 3 and 60, respectively. The corresponding carrier frequency is obtained by mapping the selected scale to the frequency and converting it to an angular frequency

(9) $${\hat{f}}_{k}\left(y\right)=f\left(\alpha_{k,y}^{*}\right),\;\omega_{k}\left(y\right)=2\pi {\hat{f}}_{k}\left(y\right)$$

where $\omega_{k}$ provides a robust prior for the carrier in the EKF prediction model for row *y*.

For each fringe pattern, a WAEKF recursion is performed along the spatial index. The state vector of 3D measurement is defined as

(10) $$X_{k}\left(x,y\right)=\left[\begin{array}{c} \omega_{k}\left(x,y\right)\\ C_{k}\left(x,y\right)\\ S_{k}\left(x,y\right) \end{array}\right]$$

where $\omega_{k}$ denotes the spatial carrier frequency. $C_{k}$ and $S_{k}$, respectively, represent the quadrature components of the fringe patterns:

(11) $$C_{k}\left(x,y\right)=B\left(x,y\right)\cos\left(\Phi_{k}(x,y)\right),\;\;\;S_{k}\left(x,y\right)=B\left(x,y\right)\sin\left(\Phi_{k}(x,y)\right)$$

where Φ(x,y) denotes the instantaneous phase of the fringe pattern at pixel.

The spatial carrier frequency is acquired by Equation (10), and the proposed process model can be represented as

(12) $$X_{k+1}=f(X_{k})+w_{k},\;\;q_{k}∼N\left(0,Q_{k}\right)$$

and $w_{k}$ denotes the process noise. In the EKF framework, the process noise covariance $Q_{k}$ characterizes the state uncertainty arising from reflectivity fluctuations, environment influences, and unmodeled disturbances during the shape measurement.

After removing the background, the measurement model can be represented as

(13) $$z_{k}\left(x,y\right)=h\left(X_{k}\right)+v_{k}$$

where $v_{k}$ is measurement noise with variance. The corresponding Jacobian matrix is

(14) $$H_{nk}=\frac{dh(X_{k})}{dX_{k}}=[0\;1\;0]$$

The EKF recursion is performed pixel by pixel; time prediction is

(15) $${\hat{X}}_{k+1}^{-}=f({\hat{X}}_{k}^{+})$$

(16) $$P_{k+1}^{-}=F_{k}P_{k}{F_{k}}^{T}+Q_{k}$$

where $F_{k}$ is the Jacobian of $f\left(\cdot \right)$:

(17) $$F_{k}=\frac{\partial f\left({\hat{X}}_{k}\right)}{\partial {\hat{X}}_{k}}=\left[\begin{array}{ccc} 1 & 0 & 0\\ -(C_{k}\sin\omega_{k}+S_{k}\cos\omega_{k}) & \cos\omega_{k} & -\sin\omega_{k}\\ \left(C_{k}\cos\omega_{k}+S_{k}\sin\omega_{k}\right) & \sin\omega_{k} & \cos\omega_{k} \end{array}\right]$$

Measurement noise matrix is adaptively adjusted using the measured and predicted values, which is defined as

(18) $$\gamma_{k+1}\left(x,y\right)=I_{k+1}^{\prime }-{\hat{X}}_{k+1}^{-}$$

(19) $$R_{k+1}=\beta R_{k}+\left(1-\beta \right)\left(\gamma_{k+1}\gamma_{k+1}^{T}+H_{k+1}P_{k+1}^{-}{H_{k+1}}^{T}\right)$$

where $\gamma_{k}$ is the innovation. $R_{k+1}$ is updated by forgetting factor $\beta \in \left(0,1\right]$. This parameter regulates the balance between historical noise information and current measurement innovation.

Measurement update of the proposed method is

(20) $$K_{k+1}=P_{k+1}^{-}H_{k+1}^{T}{\left(H_{k+1}P_{k+1}^{-}H_{k+1}^{T}+R_{k+1}\right)}^{-1}$$

(21) $${\hat{X}}_{k+1}^{+}={\hat{X}}_{k+1}^{-}+K_{k+1}\left(I_{k+1}^{\prime }-h\left({\hat{X}}_{k+1}^{-}\right)\right)$$

(22) $$P_{k+1}^{+}=P_{k+1}^{-}-K_{k+1}H_{k+1}P_{k+1}^{-}$$

The Rauch–Tung–Striebel (RTS) smoother is applied during a backward pass to refine the EKF results. By propagating information from future measurements, the RTS recursion reduces the posterior error covariance at each time step and yields a more temporally consistent state estimate. The RTS method is inherently noncausal and therefore requires offline batch processing. The wrapped phase can be performed as

(23) $${\hat{\Phi }}_{k}=\arctan\frac{{\hat{X}}_{k}\left(3\right)}{{\hat{X}}_{k}\left(2\right)}$$

### 2.3. Phase Shift Estimation of Wavelet-Assisted Adaptive EKF

Once the wrapped phases of fringe patterns are available, the phase shift deviations between successive fringe patterns can be calculated. The estimated actual phase shift can be calculated as

(24) $$\left\{\begin{array}{l} {\hat{\delta }}_{1}=0\\ {\hat{\delta }}_{2}={\hat{\Phi }}_{2}-{\hat{\Phi }}_{1}\\ {\hat{\delta }}_{3}={\hat{\Phi }}_{3}-{\hat{\Phi }}_{1} \end{array}\right.$$

According to Equations (25) and (26), the unknown background intensity and the quadrature phase components with modulation can be computed as [40]

(25) $$\left[\begin{array}{c} \hat{A}\left(x,y\right)\\ \hat{D}\left(x,y\right)\\ \hat{M}\left(x,y\right) \end{array}\right]={\left[\begin{array}{ccc} N & \sum \cos{\hat{\delta }}_{n} & \sum \sin{\hat{\delta }}_{n}\\ \sum \cos{\hat{\delta }}_{n} & \sum \cos^{2}{\hat{\delta }}_{n} & \sum \cos{\hat{\delta }}_{n}\sin{\hat{\delta }}_{n}\\ \sum \sin{\hat{\delta }}_{n} & \sum \cos{\hat{\delta }}_{n}\sin{\hat{\delta }}_{n} & \sum \sin^{2}{\hat{\delta }}_{n} \end{array}\right]}^{-1}\left[\begin{array}{c} \sum I_{k}\\ \sum I_{k}\cos{\hat{\delta }}_{n}\\ \sum I_{k}\sin{\hat{\delta }}_{n} \end{array}\right]$$

where $\hat{A}\left(x,y\right)$, $\hat{D}\left(x,y\right)$ and $\hat{M}\left(x,y\right)$, respectively, represent the background intensity, numerator and denominator. The high-frequency wrapped phase of the measured object is

(26) $${\hat{\phi }}_{h}\left(x,y\right)=\arctan\frac{\hat{D}\left(x,y\right)}{\hat{M}\left(x,y\right)}$$

Once the wrapped phase is acquired, the dual-frequency fringe patterns are employed to unwrap it and retrieve the continuous phase. Finally, the 3D shape of the measured object is accurately reconstructed.

### 2.4. Framework of the Proposed WAEKF Method

**Figure 2:**
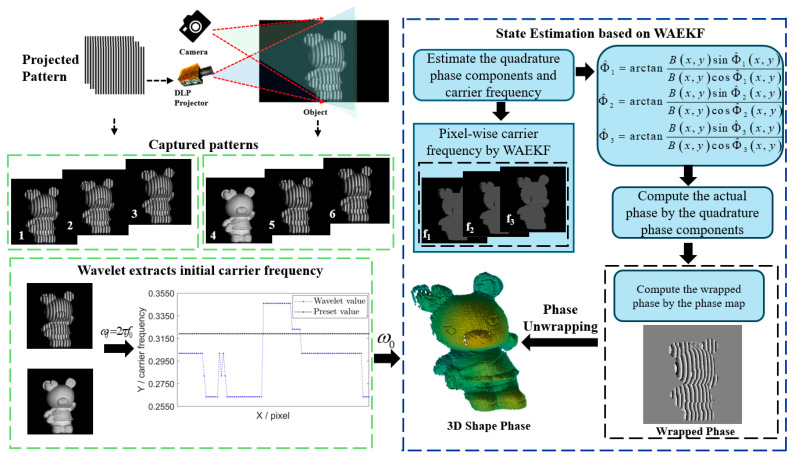
Framework of the proposed method.

A WAEKF algorithm is proposed to estimate actual phase shifts on a pixel basis in dynamic scenes. The framework of the proposed method is illustrated in Figure 2.

The framework of the WAEKF algorithm is described as follows:

Step 1: High-frequency and low-frequency fringe patterns of the measured object are captured. Based on the obtained high-frequency fringe patterns, the appropriate initial carrier frequency is acquired by the wavelet method. The extracted initialization of the carrier frequency could assist the EKF to get more accurate phase shift.

Step 2: The WAEKF method is built on the phase state-space model. The carrier frequency, the cosine and sine components are used as the state vector. Subsequently, the WAEKF is utilized to obtain the forward state estimates. The RTS smoother refines the forward state estimates during a backward process. Subsequently, the highly accurate wrapped phase is extracted from these smoothed components.

Step 3: The wrapped phase estimated by step 2 is used to calculate the actual phase shift map. The actual phase shift map can utilize Equation (25) to compute the accurate wrapped phase and eliminate motion errors by the least-squares phase shift method.

Step 4: The continuous phase is retrieved from the accurate wrapped phase by using the double-frequency three-step PSP and modified 2 + 1 PSP method [6,41,42], thereby achieving high-accuracy 3D shape reconstruction

## 3. Experiment and Discussion

### 3.1. Experimental Setup

A structure-light projection system, which consists of a camera (BaumaVCXU-31M, 1280 × 800 pixels) and a DLP projector (Light Crafter 4500), is constructed to evaluate the WAEKF method for motion objects. Translation and rotation motion are considered to verify the effectiveness and robustness of the method.

**Figure 3:**
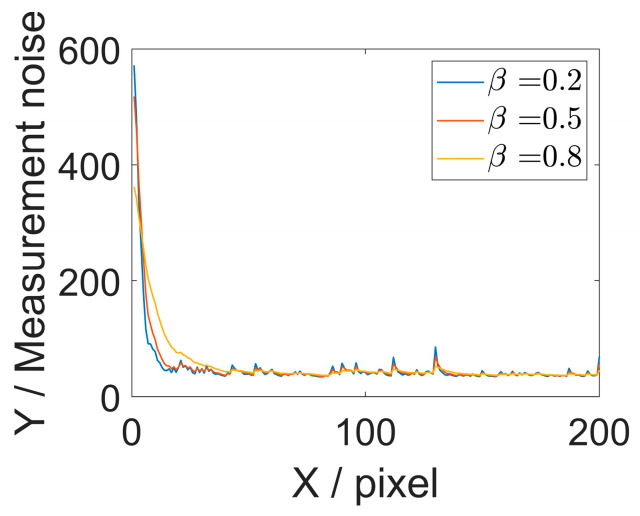
Measurement noise under different forgetting factors.

Initial parameters critically influence estimation outcomes and improper initialization setting often leads to failure. The initial modulation and covariance matrices are adaptively determined by analyzing the grayscale intensity of the fringe patterns. The forgetting factor β is set to 0.5 to balance the current measurement observation and prior estimate. Figure 3 illustrates the variations of the measurement noise under different forgetting factors. The initial experimental parameters are listed in Table 1. The value of $\omega_{0}$ is obtained by the wavelet method.

### 3.2. Experiment of Translation Motion

The experiment on translation motion of the measured object using the WAEKF method was conducted with the improved dual-frequency heterodyne method. The high frequency was 64 and low frequency was 56. The beat frequency between them was 8.

**Figure 4:**
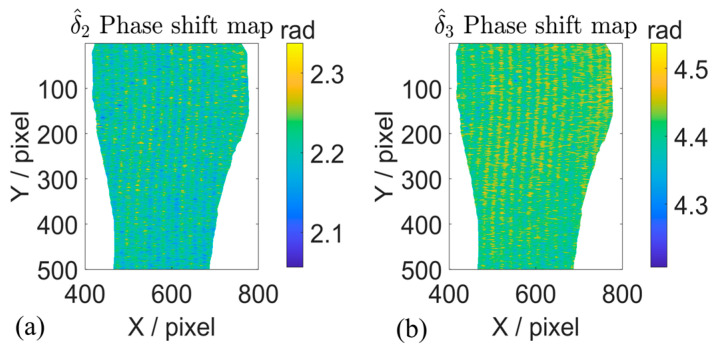
Estimated actual phase shift under the translation motion. (**a**,**b**) Three-step phase shift map estimated by the proposed method.

**Figure 5:**
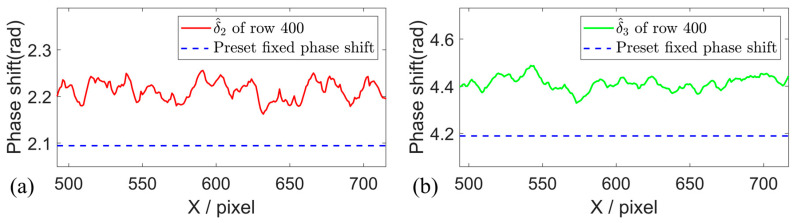
Estimated phase shift of the 400th row. (**a**,**b**) Phase shift in the 400th row estimated by the proposed method.

In the translation motion experiment, the actual phase shifts are estimated by using the proposed method, and the phase shift maps of the motion object are given in Figure 4a,b. The phase shift steps are estimated row by row and pixel by pixel within the mask region. The actual phase shift steps of row 400 are illustrated in Figure 5. The estimated phase shifts between adjacent fringe patterns vary within the range of approximately 2.2–2.3 rad and 4.3–4.5 rad, and distinctly deviate from the preset value of 2π/3 rad and 4π/3 rad in the three-step PSP. It can be observed that the fixed phase shift values have been changed in the actual measurement due to the object motion.

**Figure 6:**
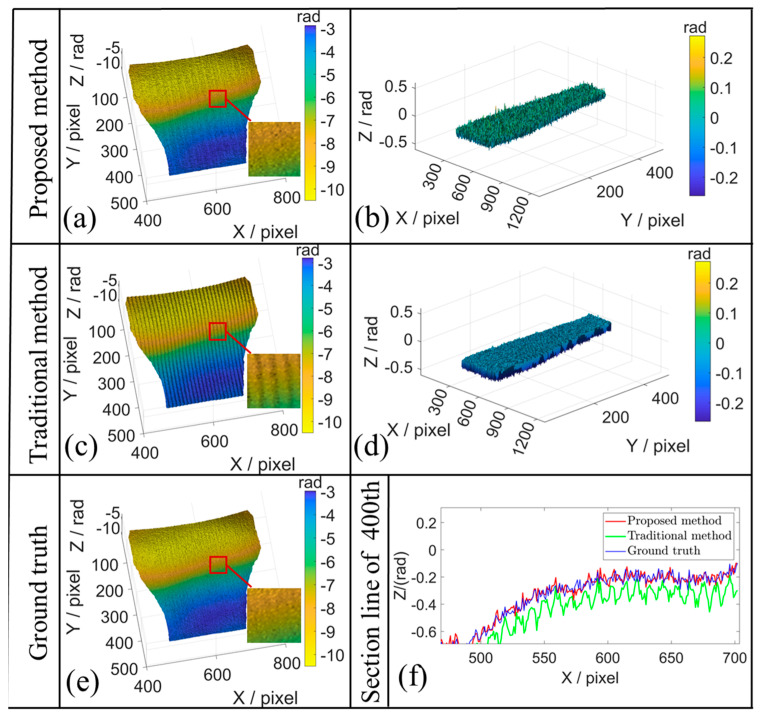
Translation experimental results of the proposed method and traditional method. (**a**,**c**) Reconstruction results of the proposed method and traditional method. (**b**,**d**) Corresponding error distribution. (**e**) Ground truth of 3D reconstruction. (**f**) Section line in the 400th row.

**Figure 7:**
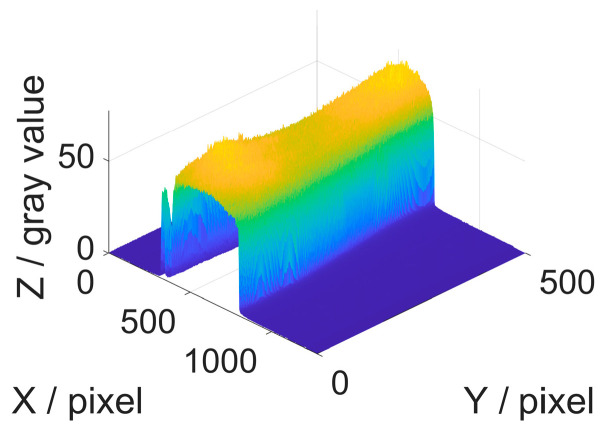
Modulation map of the translation motion object.

The comparison of the traditional three-step PSP method is shown in Figure 6 and Table 2. The 3D reconstruction results and error distribution under the translation motion using the proposed method and tradition method are presented in Figure 6a–f. It can be seen from Figure 6c that the traditional method exhibits significant motion-induced errors in the reconstructed shape, whereas the proposed method substantially mitigates the errors. The sectional line along the 400th row shown in Figure 6f indicates that the reconstructed phase using the WAEKF method closely approximates the ground truth, whereas the traditional method exhibits noticeable deviations. Figure 7 presents the modulation map to analyze the impact of low SNR. To eliminate the impact of low SNR, a modulation threshold mask is applied. By excluding the masked low-SNR regions from the EKF processing, the denominator in Equation (19) is prevented from approaching zero, thereby effectively avoiding numerical divergence. The evaluation metrics are shown in Table 2. The WAEKF approach demonstrates significant improvements in the root-mean-square error (RMSE) and standard deviation (STD) relative to the traditional method. It demonstrates that the proposed approach eliminates motion-induced phase errors and yields an accurate reconstruction.

### 3.3. Experiment of Rotation Motion

**Figure 8:**
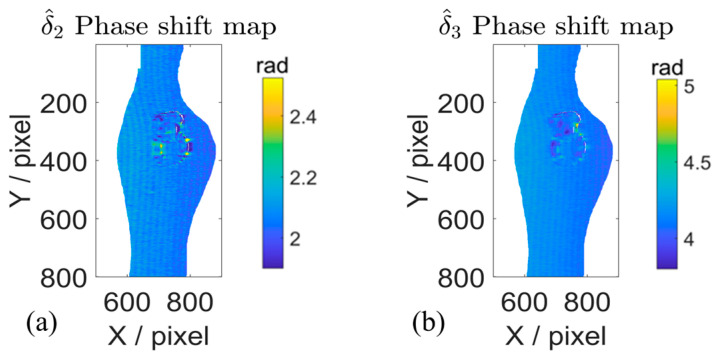
Estimated actual phase shift under the rotation motion. (**a**,**b**) Three-step phase shift map estimated by the proposed method.

**Figure 9:**
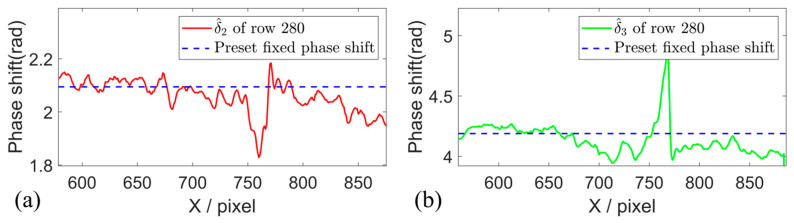
Estimated actual phase shift of the 280th row. (**a**,**b**) Phase shift in the 280th row estimated by the proposed method.

To further validate the effectiveness of the WAEKF method, the rotation motion experiments were conducted, and a Fourier-assisted approach (FAPS) [19] is used for comparison. The estimated phase shift using the WAEKF method is presented in Figure 8. The results demonstrate that the proposed algorithm accurately estimates the actual phase step of the captured fringe. The actual phase shifts of the 280th row are shown in Figure 9.

**Figure 10:**
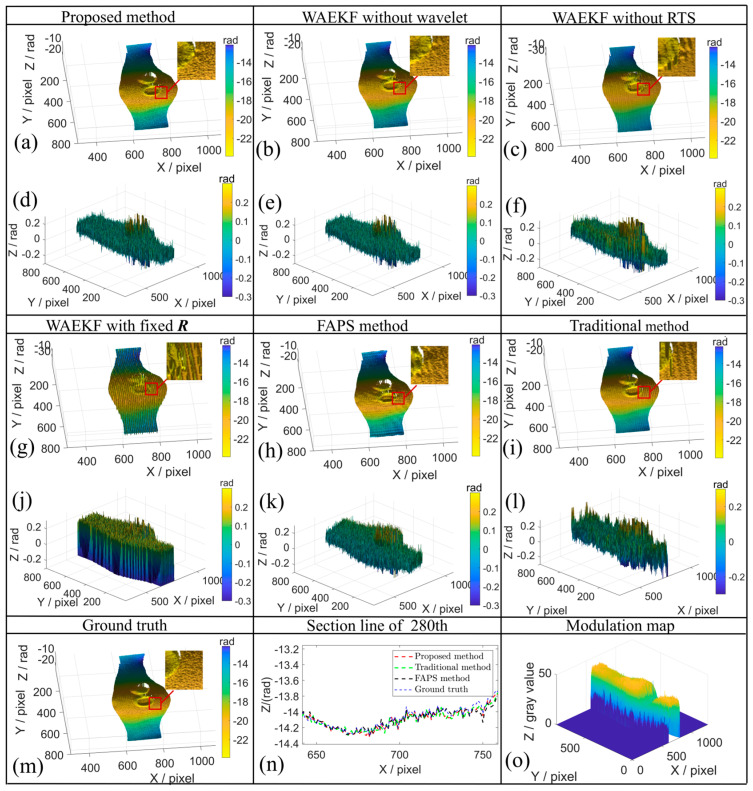
Rotation experiment results, which include 3D reconstruction and error distribution of different methods. (**a**,**d**) Proposed method. (**b**,**e**) WAEKF without wavelet. (**c**,**f**) WAEKF without RTS. (**g**,**j**) WAEKF with fixed ***R.*** (**h**,**k**) FAPS method. (**i**,**l**) Traditional method. (**m**) Ground truth of 3D reconstruction. (**n**) The section line in the 280th row. (**o**) Modulation map of the rotation motion object.

The 3D reconstruction results are illustrated in Figure 10. The 3D shape of the measured object is recovered using different methods, as depicted in Figure 10a–c,g–i,m. The proposed method effectively suppresses motion-induced artifacts caused by rotation motion and yields a smoother reconstructed shape compared with the traditional method. This indicates that the proposed algorithm is reliable and robust to the rotation motion. The error distribution maps and comparison results are shown in Figure 10d,e,j–l; it can be seen from that the retrieved phase using the proposed method is in close agreement with the ground truth. To analyze the impact of a low SNR during rotation, Figure 10o presents the corresponding modulation map. Consistent with the translational case, applying the threshold mask excludes these low-SNR regions from the EKF processing, effectively preventing numerical divergence. Figure 10n indicates that the section line of the proposed method is closer to the ground truth. Experimental results demonstrate that even in regions with significant deformation, the proposed method maintains reliable estimation performance and yields improved overall reconstruction accuracy.

As summarized in Table 3, the WAEKF method significantly outperforms the traditional method and yields smaller errors than the FAPS method. More importantly, ablation experiments clearly validate the indispensable role of each algorithm. Quantitatively, compared to the optimal RMSE of 0.0446 rad, the error increases to 0.0766 rad without wavelet initialization and to 0.0568 rad without RTS smoothing in the WAEKF. Notably, the method with fixed ***R*** achieves the highest RMSE of 0.2066 rad, directly confirming the critical necessity of the adaptive mechanism. The comparative evaluation confirms the optimization achieved by the wavelet, RTS and adaptive ***R*** components. Furthermore, the results indicate that the adaptive ***R*** provides a better improvement in WAEKF. It demonstrates that all modules are indispensable. Similarly, the identical conclusion is evident in STD values. Experimental results demonstrate that the proposed method is effective for both translation and rotation motions.

**Figure 11:**
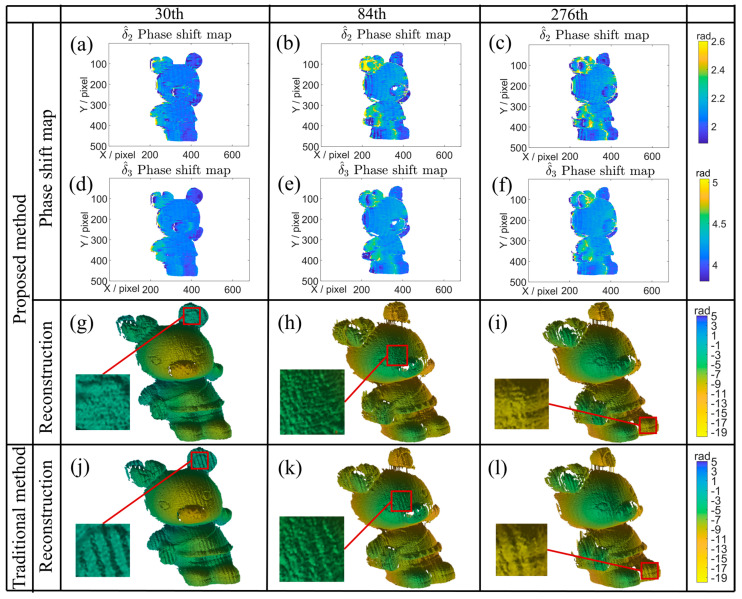
3D reconstruction results of successive frames. (**a**–**f**) Phase-shift map obtained by the proposed method. (**g**–**i**) 3D reconstruction using the proposed method at frames 30, 84, and 276, respectively. (**j**–**l**) 3D reconstruction using the traditional method at frames 30, 84, and 276, respectively.

To further verify its performance under the rotation motion, an additional experiment is conducted. The phase shift maps and 3D reconstructed results of the rotation experiment are presented in Figure 11. As observed in Figure 11a–f, the WAEKF method effectively captures the spatial phase shift derivation introduced by the rotation motion. The estimated phase shift values fall within the range of approximately 2–2.6 rad and 4–4.6 rad, and their spatial distribution varies according to the motion state and shape geometry of the measured object. The accurate phase shift estimation leads to noticeably accurate reconstructed phases shown in Figure 11g–i. In contrast, the traditional method produces motion artifacts in Figure 11j–l. The traditional method is unable to compensate for spatial phase errors under rotation, so the reconstructed 3D shape exhibits local motion-induced ripples. By comparison, the proposed approach offers more reliable and accurate reconstruction results, with a clear reduction in motion ripples induced by the object’s movement. These experiments verify that the WAEKF-based motion error compensation method enhances the accuracy in dynamic 3D shape measurement.

**Figure 12:**
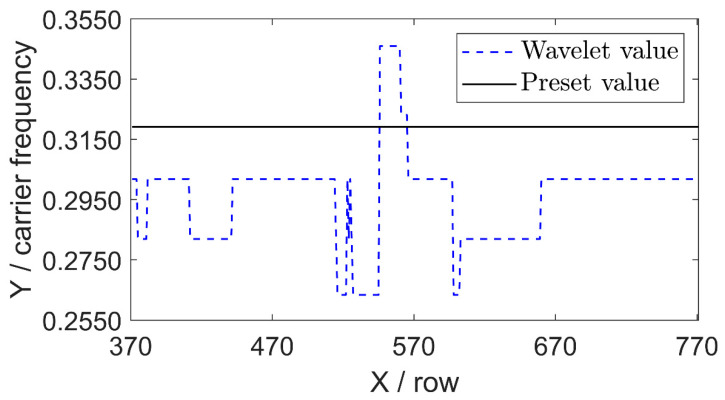
Initial carrier frequency rows 330 to 770 at the 30th frame using the wavelet-assisted method.

Before EKF, the initial carrier frequency per row obtained from the wavelet method is shown in Figure 12. The initial carrier frequency has significant deviation in different rows, indicating that the actual carrier frequency varies spatially under the object motion. The extracted initial carrier frequency serves as robust prior information for the WAEKF method and facilitates accurate phase recovery in the subsequent EKF.

### 3.4. Discussion

The proposed framework provides a feasible and effective solution for addressing motion-induced errors in dynamic measurements, while several aspects still require further investigation. It should be noted that the current framework performs motion error compensation in an offline manner during the 3D reconstruction process, which limits its real-time applicability. In addition, the robustness of the method under high-speed motion conditions remains to be further explored. Moreover, highly complex object geometries or abrupt depth discontinuities may still affect the stability of fringe analysis and degrade the reconstruction accuracy. Future work will focus on further improving system performance through the integration of advanced high-speed projectors and cameras, enhanced real-time capability, and the background optimization, so as to further reduce motion artifacts caused by moving objects.

## 4. Conclusions

A pixel-wise phase shift estimation method based on a wavelet-assisted adaptive EKF is presented to address the motion-induced phase errors in 3D reconstruction measurement. A state-space model is constructed using the quadrature components and carrier frequency in this work. The wavelet-assisted method can extract the initial carrier frequency from the fringe patterns, which is used as a prior to constrain the adaptive EKF for pixel-wise estimation. The proposed estimation framework derives the wrapped phases from the WAEKF-estimated quadrature components, and the inter-frame phase shift deviations are evaluated between successive patterns to achieve the accurate 3D shape. Experiments demonstrate that the phase errors caused by the object motion are effectively reduced and the reconstruction accuracy in dynamic measurements is enhanced by the proposed WAEKF method.

This method offers a practical reference for 3D sensing in industrial environments and biomedical imaging where motion is unavoidable. For instance, it is applicable to biomedical imaging, such as dental orthodontics; by compensating for phase errors caused by inevitable device and head movement during scanning, the system can retrieve more accurate 3D dental morphology without requiring absolute patient immobility. Ultimately, the proposed WAEKF framework provides a robust solution for high-accuracy 3D reconstruction in scenarios involving motion.

## Figures and Tables

**Table 1:** Parameter setting of the WAEKF algorithm.

Algorithm Parameters	
$P_{0|0}=diag\left(\left[1e-6,600,600\right]\right)$	$X_{0}={\left[\omega_{0},B\cos(\pi /6+\pi /10),B\sin(\pi /6+\pi /10)\right]}^{T}$
$Q_{0}=diag\left(\left[1e-10,1e1,1e1\right]\right)$	$B=\frac{1}{2}\left[\max\left(I_{k}\right)-\min\left(I_{k}\right)\right]$
$R_{0}=9$	β=0.5

**Table 2:** Comparison of 3D reconstruction results with different methods under translation motion.

	RMSE (Rad)	STD (Rad)
The WAEKF method	0.0338	0.0338
The traditional method	0.1258	0.0569

**Table 3:** Comparison of 3D reconstruction results with different methods under rotation motion.

	RMSE (Rad)	STD (Rad)
The WAEKF method	0.0446	0.0446
The WAEKF without wavelet	0.0766	0.0766
The WAEKF without RTS	0.0568	0.1152
The WAEKF with fixed ***R***	0.2066	0.1211
The FAPS method	0.0708	0.0707
The traditional method	0.1048	0.1030

## Data Availability

The data presented in this study are available from the corresponding author upon reasonable request.
